# Response of Three *Miscanthus* × *giganteus* Cultivars to Toxic Elements Stress: Part 2, Comparison between Two Growing Seasons

**DOI:** 10.3390/plants11070945

**Published:** 2022-03-30

**Authors:** Karim Suhail Al Souki, Clarisse Liné, Jiří Moravec, Francis Douay, Bertrand Pourrut

**Affiliations:** 1 Department of Environmental Chemistry and Technology, Faculty of Environment, Jan Evangelista Purkyně University in Ústí nad Labem, Pasteurova 3632/15, 400 96 Usti nad Labem, Czech Republic; karim.souki@ujep.cz; 2 Laboratoire Génie Civil et géo-Environnement (LGCgE), ISA Lille, Junia, 48 Boulevard Vauban, CEDEX, F-59046 Lille, France; clarisse.line@ensat.fr (C.L.); francis.douay@junia.com (F.D.); 3 Laboratoire Écologie Fonctionnelle et Environnement (ECOLAB), Université de Toulouse, CNRS, INPT, UPS-ENSAT, Avenue de l’Agrobiopôle, F-31326 Castanet-Tolosan, France; 4 Department of the Environment, Faculty of Environment, Jan Evangelista Purkyně University in Ústí nad Labem, Pasteurova 3632/15, 400 96 Usti nad Labem, Czech Republic; jiri.moravec@ujep.cz

**Keywords:** *Miscanthus* × *giganteus*, TE contamination, pot experiment, antioxidative response

## Abstract

The positive impact on restoring soil functionality, decreasing toxic elements (TE) bioaccessibility, and enhancing soil physicochemical and biological parameters established a consensus on considering a *Miscanthus* × *giganteus* convenient species for phytomanaging wide TE contaminated areas. Nevertheless, information about the plant’s mode of reaction to elevated soil multi-TE concentrations is still scarce. For the sake of investigating the miscanthus response to stressful TE concentrations, an ex-situ pot experiment was initiated for 18 months, with three miscanthus cultivars referred to as B, U, and A planted in soils with gradient Cd, Pb, and Zn concentrations. A non-contaminated control soil was introduced as well, and plants were cultivated within. Results revealed that the long exposure to increasing soil TE concentrations caused the number of tillers per plant to decline and the TE concentrations in the leaves to boost progressively with the soil contamination. The photosynthetic pigments (chlorophyll a, b, and carotenoids) were negatively affected as well. However, the phenolic compounds, flavonoids, tannins, and anthocyanins, along with the antioxidant enzymatic activities of superoxide dismutase, ascorbate peroxidase, and glutathione reductase elevated progressively with the TE concentration and exposure duration. Conclusively, miscanthus plants demonstrated an intensified and synchronized antioxidative activity against the TE concentration.

## 1. Introduction

Considerable areas of agricultural lands in the proximity of mining and smelting sites are vulnerable to contamination by multiple toxic elements (TE) as a result of the atmospheric emissions and deposition of dust particles [1]. TE accumulation in agricultural soils is of concern due to food safety issues, potential health risks, as well as detrimental effects on soil ecosystems [2]. The areas surrounding the former Pb smelter Metaleurop Nord in Northern France are an explicit example of the complexities that might arise as a result of intense land TE pollution [3,4]. The former smelter was active for more than a century and generated great quantities of dust. Consequently, the agricultural topsoils in the vicinity of the smelter are extremely contaminated by TE (mainly Cd, Pb, and Zn), and the agricultural food crops might not meet the European standards [3]. In addition, several human disease dysfunctions have been documented in the inhabitants as a result of their chronic exposure to the corresponding metals [3,5,6]. Therefore, sustainable management of these soils is crucial. 

The conventional physicochemical remediation methods are not convenient for treating vast areas, due to their high costs and negative impacts on the ecological system. Therefore, environmentally friendly and cost-effective biotechnologies capable of reducing risks while enhancing the ecological quality of the polluted areas are under investigation [7,8]. The most appropriate alternative technology for the vast multi-contaminated area surrounding the former Metaleurop Nord smelter is in-situ phytostabilization. Upon the application of phytostabilization, TE leaching through polluted soil is reduced, as well as their corresponding availability [6]. Moreover, TEs are sequestered in the roots and their translocation and accumulation in the aboveground parts of the plant are limited [6]. In the scope to find the most appropriate technique for reclaiming the large, contaminated area, several plants species were trialed. The herbaceous ryegrass (*Lolium perenne*) and white clover (*Trifolium repens*) were first selected for their ability to form a dense plant cover on the soil, thus limiting soil erosion and dust emissions [9,10]. These plants were able to limit the TE transfer to their aerial parts over time and were relatively tolerant of the stressors, making them suitable for phytostabilization. Meanwhile, the efficiency of aided-phytostabilization combining the use of five woody species (*Robinia pseudoacacia*, *Alnus glutinosa*, *Quercus robur*, *Acer pseudoplatanus*, and *Salix alba*) and fly ash amendment was also evaluated [11,12,13]. The results demonstrated successful afforestation of the site, combined with the reduction in TE availability, especially in the remediated plots. However, the economic interest of these crops is rather limited, which restrained in interest in them for the phytomanagement of a contaminated area as large as Metaleurop. 

In this specific context, the most promising phytomanagement option appeared to be the cultivation of *Miscanthus* × *giganteus* [14,15]. This plant is a C4 perennial herbaceous, lignocellulosic, rhizomatous, and noninvasive grass, capable of producing high yield, adapting to high TE, organic, and/or a mixture of contaminants, as well as enhancing soil carbon sequestration [16,17,18]. Due to its high capacity to accumulate TE mainly in roots and thus reduce their potential mobility and bioavailability, miscanthus plants contribute to alleviating the human and environmental risks [14]. Several papers have been released reporting the positive impacts of miscanthus on degraded areas such as Metaleurop Nord. Of which, a study demonstrated its high capacity for accumulating low TE and nutrient concentrations in the aerial parts and proliferation in highly contaminated agricultural plots where it acquired considerable dry biomass which could be valorized economically [15,19]. Pelfrêne et al. [6] demonstrated its substantial impacts on TE redistribution in soils as well as on decreasing human metal oral bioaccessibility. Finally, Al Souki et al. [20] displayed the positive impacts of the *Miscanthus* × *giganteus* in restoring TE contaminated soil functionality via enhancing the soil biological activities, microbial biomass carbon, and certain physicochemical parameters, such as the soil organic carbon and cationic exchange capacity. Nevertheless, the vast majority of the published papers represent experiments that were conducted in non-realistic growing conditions (hydroponics, spiked soils with elevated TE concentrations, or substrates that do not represent real soil parameters). Thus, there is still much knowledge lacking at the level of TE exposure and accumulation effects on plant health and resistance mechanisms [21,22,23]. Moreover, the publications that came out discussed the TEs’ impacts on miscanthus for a period of between three and four months or at a maximum, during one growing cycle. Thus, there is a need to understand the plant’s tolerance and resistance mechanisms against TEs.

Within this context, the current work is part of a larger study aimed at understanding the influence of *Miscanthus* × *giganteus* on soil parameters [4,20] and its tolerance to TEs during two growing seasons [24]. In this study, we investigated the response of *Miscanthus* × *giganteus* plants cultivated in pots filled with soils from the Metaleurop area with a gradient concentration of Cd, Pb, and Zn, after 18 months of cultivation (two growing periods). We studied the effects of soil TE contamination on three different cultivars’ (i) plant growth parameters and accumulation of Cd, Pb, and Zn in leaves, (ii) oxidative stress induction in leaves following antioxidant enzyme activities, (iii) secondary metabolism (phenolic compounds, tannins, flavonoids, and anthocyanins) and photosynthetic pigments (chlorophyll a, b, and carotenoids).

## 2. Materials and Methods

### 2.1. Pot Experiment Preparations 

As previously mentioned, the agricultural land surrounding the former Pb smelter Metaleurop Nord is massively contaminated by several TEs [10,25]. Nevertheless, the choice of focusing on Cd, Pb, and Zn was made due to their elevated concentrations in the soils, the presence of proven statistical correlation between these TEs, the familiarity of working with these elements in our laboratory, and finally, due to the fact that this work represents a continuity of a previous one that studied particularly these 3 TEs. Soil samples (plowed horizon, 0–25 cm) were collected from different agricultural plots in the territory surrounding the former smelter and characterized by their gradient TE concentrations which increased as the distance to the smelter decreased [3,25]. Samples were thereby designated based on their Pb concentration (in mg kg^−1^ in soil), in which M200 (50°24′52″ N, 3°01′51″ E, Courcelles-les-Lens, 1.8 km from the smelter, 1.4 ha), M500 (50°25′49″ N, 3°02′13″ E, Evin-Malmaison, 1.4 km from the smelter, 0.8 ha), M750 and M900 (50°26’15.0′′ N 3°01’05.7′′ E, Evin-Malmaison, 1 km from the smelter, 0.8 ha) samples contained approximately 200, 500, 750, and 900 mg kg^−1^ Pb in soil. For the sake of comparing the impact of contamination on miscanthus, soil samples were collected from a non-contaminated agricultural plot located 75 km from the smelter (50°20′46″ N 2°12′15″ E, Linzeux, 1.3 ha) and considered as controls. Thereafter, samples were homogenized, dried, and sieved through a 10 mm mesh prior to cultivation.

Three different miscanthus cultivars (B, U, and A) from different origins were used in the pot experiment. Small 2–3 budded rhizomes (5–7 cm) of the corresponding plants were grown in pots (9 × 9 × 9 cm) filled with potting compost that was kept wet via constant watering.

### 2.2. Pot Experimental Design

When the miscanthus plantlets reached 20–25 cm height, an ex-situ experiment was launched for a period of 18 months (May 2014 to October 2015) in an area distant from roads on the University of Lille campus (50.6090° N, 3.1381° E). Approximately 100 kg of the upper mentioned homogenized and dry soils (MC, M200, M500, M750, and M900) were equally distributed in five 20-kg capacity pots (light grey colored in order to limit temperature elevation possibilities) and the *M.* × *giganteus* plantlets (B, U, and A) were thereafter displaced from the small pots to the 20-kg soil pots (two plantlets in each pot).

Table 1 represents the TE concentrations in the studied soils. As previously shown by Al Souki et al. [20,24], the TE concentrations in MC were in agreement with the regional background values (0.42, 38, and 74 mg kg^−1^, corresponding to Cd, Pb, and Zn, respectively). Whereas the other soils displayed pseudo total concentrations 20 to 50 times higher than the regional agricultural background values.

Briefly, 75 planted pots were used (3 different miscanthus cultivars × 5 soils × 5 replicates). The pots were placed over wooden rafters to avoid direct contact with the experimental ground area. Random distribution of the pots took place in an attempt to avoid point and borderline effects. Moreover, the soils were regularly irrigated during the entire experiment to sustain the soils’ humidity, along with rainwater. Weeds were manually removed and kept on the soils’ surface to avoid possible TE exportation.

### 2.3. Miscanthus Sampling and Preparation

By the end of the second growing season (in October), plant growth parameters, such as the stem height, diameter, and number of tillers per pot, were determined for the second time.

For the sake of investigating the contamination effects on plants’ health, three leaves (4th, 5th, and 6th foliar stage) were collected from every plant. The samples were instantly flash-frozen in liquid nitrogen and then conserved at −80 °C in the laboratory prior to biomarker analysis.

The rest of the leaves within each pot were also collected to determine their TE concentrations. Samples were placed in a plastic bag and kept in a cool box. When in the laboratory, the leaves were washed 3 times with osmosed water in order to eliminate the dust particles. Later on, samples were oven-dried at 40 °C for 48 h and ground into a fine powder using a knife mill (GM200, Retsch) before TE analysis.

#### 2.3.1. TE Concentrations in the Collected Leaves

Cd, Pb, and Zn concentrations in miscanthus leaves were determined via acid digestion. Three hundred milligram of the ground leaf powder was digested with nitric oxide (HNO_3_ 70%) and heated. Afterward, hydrogen peroxide (H_2_O_2_ 30%) was added, and the sample was reheated for another 180 min prior to osmosed water addition. The TEs in the extracts were determined by atomic absorption spectrophotometry (AA-6800, Shimadzu, Kyoto, Japan). Quality control for chemical extraction and digestion was performed by including blanks, internal, and certified (Polish Virginia tobacco leaves, INCTPVTL-6, Poland, Warsaw, Poland) reference materials [26]. 

#### 2.3.2. Antioxidant Enzymatic Activities Assays

Antioxidative enzymatic activity assays were evaluated spectrophotometrically according to Al Souki et al. [24], using a plate reader (Thermo Scientific Multiskan™ GO, Illkirch-Graffenstaden, France). Briefly, five foliar discs (0.5 cm diameter) per plant sample were collected from frozen leaves by a manual punch. Then, they were put into a 96-deep-well plate (2 mL) having one 4 mm diameter glass bead. Under frozen conditions, the samples were then ground twice by Mixer Mill MM 400 (Retsch, Haan, Germany) (1.5 min, 30 Hz). After the addition of 1 mL of ice-cold Tris extraction buffer pH 7.0 containing 0.01 M EDTA, 0.4 M PVP, 0.05 ascorbate, 11.44 mM β-mercaptoehtanol, and proteases cocktail inhibitor, samples were homogenized for 2 min at 15 Hz with the MM400 (Retsch, Haan, Germany) grinder. Plates were then centrifuged at 5000× *g* for 15 min at 4 °C. Supernatants were collected and protein content was determined according to Bradford [27], using bovine serum albumin (BSA, Sigma, Saint-Quentin-Fallavier, France) as standard.

Total superoxide dismutase (SOD) activity was determined by measuring its ability to inhibit the photochemical reduction of nitro blue tetrazolium (NBT), according to the method of Giannopolitis and Ries [28]. The reaction mixture contained 0.47 mM NBT (Sigma), 3.85 µM riboflavin (Sigma), 19.23 mM methionine (Sigma), 36.54 mM phosphate buffer (pH 7.8), and 20 µL enzyme extract. The test tubes containing the mixture were placed 30 cm below a light source (30 W fluorescent lamps). The reaction was started by switching on the light and was allowed to run for 10 min. The reaction was ceased by switching off the light with the absorbance at 560 nm. An unirradiated reaction mixture that did not develop color served as the control, and its absorbance was subtracted from that of the test tube. One unit of SOD activity was defined as the amount of enzyme required to cause 50% inhibition of NBT reduction.

Ascorbate peroxidase (APX) activity was evaluated by the decrease in absorbance at 290 nm due to ascorbate oxidation [29]. The reaction mixture contained 434 mM phosphate buffer (pH 7.0), 3.77 mM H_2_O_2_, 0.56 mM ascorbic acid, and 10 µL enzyme extract. One enzyme unit was defined as 1 µmole of ascorbic acid oxidized per min at 290 nm, using a standard curve of ascorbate. The enzyme activity was expressed as µmoles of ascorbate oxidized min^−1^ mg^−1^ protein.

Glutathione reductase (GR) was assayed as the decrease in absorbance at 340 nm caused by NADPH oxidation [30]. This assay is based on the reduction of oxidized glutathione (GSSG) by NADPH in the presence of GR. The reaction mixture contained 0.1 M Tris buffer (pH 7.5), 1 mM GSSG (Sigma), 0.1 mM NADPH (Sigma), and 20 µL of enzyme extract. The amount of NADPH oxidized was calculated from the extinction coefficient of 3.732 × 10^−3^ mL nmole^−1^ of NADPH. The enzyme activity was expressed as nmoles of NAPDH oxidized min^−1^ mg^−1^ protein.

#### 2.3.3. Photosynthetic Pigments and Secondary Metabolism Molecules Quantification

Photosynthetic pigments, phenolic compounds, tannins, flavonoids, and anthocyanins were determined spectrophotometrically, according to Al Souki et al. [24], by a plate reader (Thermo Scientific Multiskan™ GO). Briefly, two foliar discs (0.5 cm diameter) per plant sample were collected from frozen leaves using a manual punch and weighed. Then, they were put into in 96-deep-well plate (2 mL) with one 4 mm diameter glass bead. Samples were then ground under frozen conditions using a Mixer Mill MM 400 (Retsch) for 2 times 1.5 min at 30 Hz. After the addition of 1.5 mL of ice-cold 95% methanol in each well, samples were homogenized for 2 min at 15 Hz with the MM400 grinder. Plates were left in the dark for 24 h and 48 h of incubation. 

After 24 h of incubation, leaf extracts were homogenized for 2 min at 15 Hz with the Mixer Mill MM 400. One hundred microliter was collected for photosynthetic pigment analysis. The absorbance was measured at 470, 652, and 666 nm. Concentrations of chlorophyll a, b, and total carotenoids were calculated according to extinction coefficients and equations reported by Lichtenthaler [31]. Finally, data were averaged, and the obtained mean concentrations were expressed as mg g^−1^ FW of the leaf. 

After 48 h of incubation, plates were centrifuged at 5000× *g* for 5 min, prior to secondary metabolism molecule extraction. Total phenolic compounds were determined based on the Folin Ciocalteu assay. Briefly, the reaction mixture of 200 µL contained 20 µL of supernatant, 40 µL of Folin reagents (10% *v*/*v*), and 0.098 mM of Na_2_CO_3_. The mixture was allowed to stand for 2 h at room temperature for color development. Later on, the absorbance was measured at 510 nm. Concentrations of phenolic compounds were calculated using a standard curve of gallic acid. Results were expressed as mM of gallic acid equivalent (GE) per gram of fresh weight of leaf. The flavonoid content was determined by the aluminum chloride method using catechin as a reference compound. Briefly reaction mixture contained 25 µL of methanolic extract, 0.00724 mM NaNO_2_, 0.01125 mM AlCl_3_, and 0.05 mM NaOH. The mixture was homogenized for 60 sec and absorbance was measured at 595 nm. Flavonoid concentrations were calculated using a standard curve of catechin. Results were expressed as mg catechin equivalent (CE) per gram of fresh weight of leaf. For tannins determination, the reaction mixture contained 50 µL of methanolic extract and 100 µL of vanillin solution 1%. The mixture was kept in dark for 15 min and the absorbance was measured at 500 nm. Tannin concentrations were calculated using a standard curve of catechin. Results were expressed as mg L^−1^ catechin equivalent (CE) per gram of fresh weight of leaf. Finally, anthocyanins were measured using the differential pH method based on the property of anthocyanin pigments to change color with pH. Two dilutions of the same sample were prepared, one in potassium chloride buffer (0.2 M, pH 1.0) and the other in sodium acetate buffer (0.4 M, pH 4.5). Upon a 15 min period of equilibration at room temperature, the absorbance was read at 510 and 700 nm. Results were expressed as mg cyaniding 3-glucoside equivalent per gram of fresh weight of leaf.

### 2.4. Statistical Analysis

Analysis of variance was done to compare modalities. The Fisher test was considered for significance (*p* ≤ 0.05). If statistically significant differences were found, the Tukey HSD test was used for pair-wise comparisons. All statistical analyses were performed using XLSTAT software (Paris, France).

## 3. Results

### 3.1. Miscanthus Leaf TE Concentrations at the End of the Second Growing Season

The leaf TE concentrations increased progressively with their corresponding soil concentrations in which the plants were cultivated (Table 2).

Cd concentration in the leaves of the three miscanthus cultivars cultivated in the MC soil was 0.4 mg kg^−1^ and increased with the TE concentration in soil. Values were 7.8, 6.8, and 7.8 times more in B, U, and A plants cultivated in the M900 soil. 

Concomitantly, Pb concentrations in the leaves of the three cultivars in M900 were 4.5-fold their concentrations in the non-contaminated plants (6.9, 5.9, and 7.0 1 mg kg^−1^ corresponding to B, U, and A). 

Zn concentrations in the leaves increased with the soil contamination as well. Their values in the M900 soils were 3.7, 4.0, and 3.4 times more than the ones in MC soil (37.2, 29.0, and 41.5 mg kg^−1^ corresponding to B, U, and A cultivars, respectively). 

### 3.2. Stem Height, Diameter, and Number of Tillers per Plant at the End of the Second Growing Season

Table 3 presents stem height, diameter, and tillers number of the three miscanthus cultivars at the end of the second growing season in each pot of the studied soils.

The highest tillers number was observed in the three miscanthus cultivars planted in MC soil (16.3, 27.3, and 17.0 tillers per plant corresponding to B, U, and A cultivars, respectively). Significant differences occurred within the plants cultivated in the non-contaminated MC soil, in which U cultivar exhibited more tillers in the pots than B and A plants. Tillers number significantly decreased in the contaminated soils averaging between 38.8 and 72.9% compared to MC soil. However, no significant differences were displayed among the three cultivars present in the contaminated soils. 

No significant differences were detected concerning the miscanthus stem height and diameter in all the studied soils. The shoots in MC soil were slightly higher (109.3, 92.7, and 106.3 cm, corresponding to miscanthus B, U, and A, respectively). It is noteworthy to mention that the tallest U plant was shorter than the shortest plant in the other two cultivars. The thinnest stem was detected in the U plant cultivated in MC soil (7.3 mm), whereas the thickest stem belonged to B cultivar presented in MC soil as well (9.3 mm).

### 3.3. SOD, APX, and GR Activity Determination

**Figure 1:**
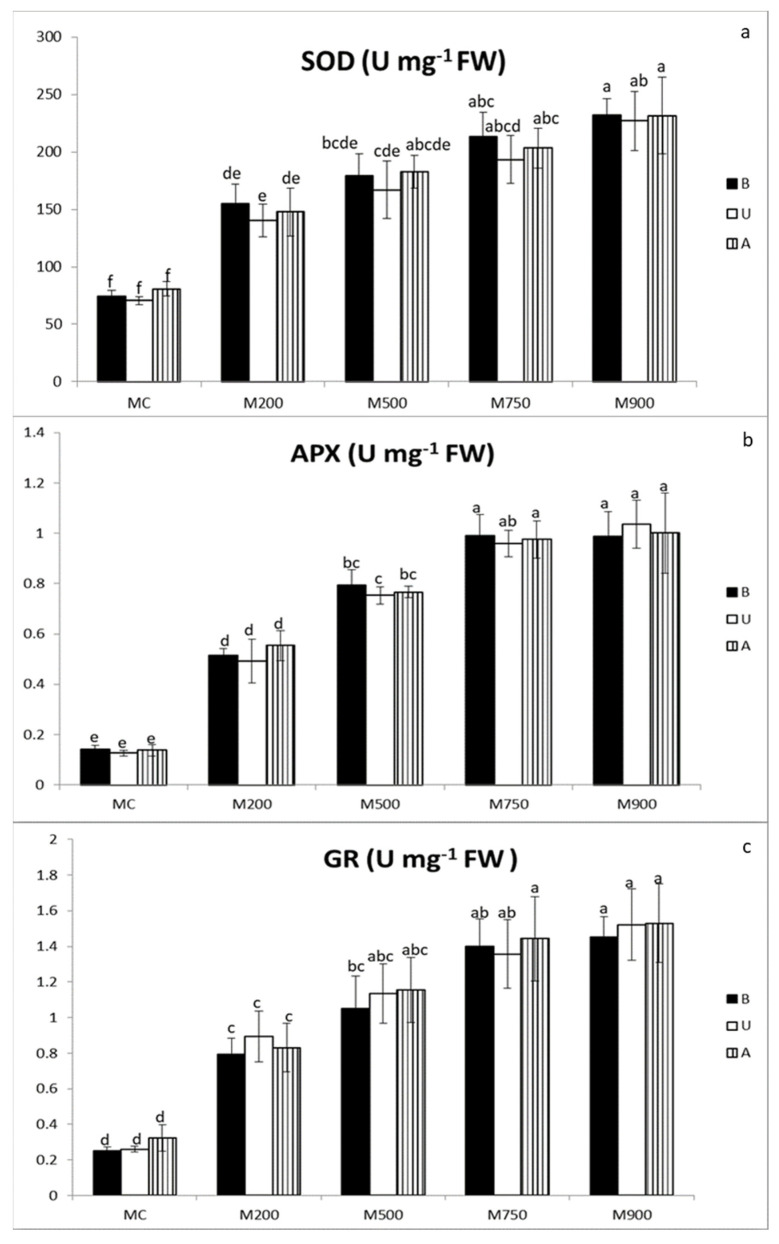
SOD (**a**), APX (**b**), and GR (**c**) activities in the leaves of three different miscanthus cultivars (B, U, and A) cultivated in soils with gradient TE concentrations (MC, M200, M500, M750, and M900). Values are presented as means ± SD. Different letters refer to significant differences between plants (Turkey HSD test, *n* = 5, *p* ≤ 0.05).

Globally, the antioxidant enzymatic activities (SOD, APX, and GR in Figure 1a, b, and c, respectively) exhibited the same response patterns to TE contamination. The enzymes were strongly activated in plants grown in M200 soil compared to MC soil. Then, their activities slowly increased, yet, in a concentration-dependent manner. For the three enzymes, no significant differences were observed among the cultivars.

The least SOD activities were obtained in the non-contaminated leaves in MC soil (74.6, 70.5, and 80.9 U mg^−1^ FW corresponding to B, U, and A, respectively), and progressively increased with the soil TE concentration, recording and accretion of 210.8, 222.0, and 186.1% in the plants in M900 soil compared to background values. 

APX minimal activities as well were recorded in the non-contaminated MC leaves (0.1 U mg^−1^ FW in the leaves of B, U, and A, respectively), and continued augmenting to reach their maximal levels in the M900 plant leaves with 602.7, 720.9, and 627.2% augmentation compared to background values.

GR recorded their lowest activities in the leaves of the plants cultivated in the MC soil (0.3 U mg^−1^ FW corresponding to B, U, and A, respectively), and increased with soil TE concentration, recording their maximal values in the plants cultivated in the M900 soil with a rise of 475.7, 485.3, and 374.9% compared to background values.

### 3.4. Photosynthetic Pigment Quantification

**Figure 2:**
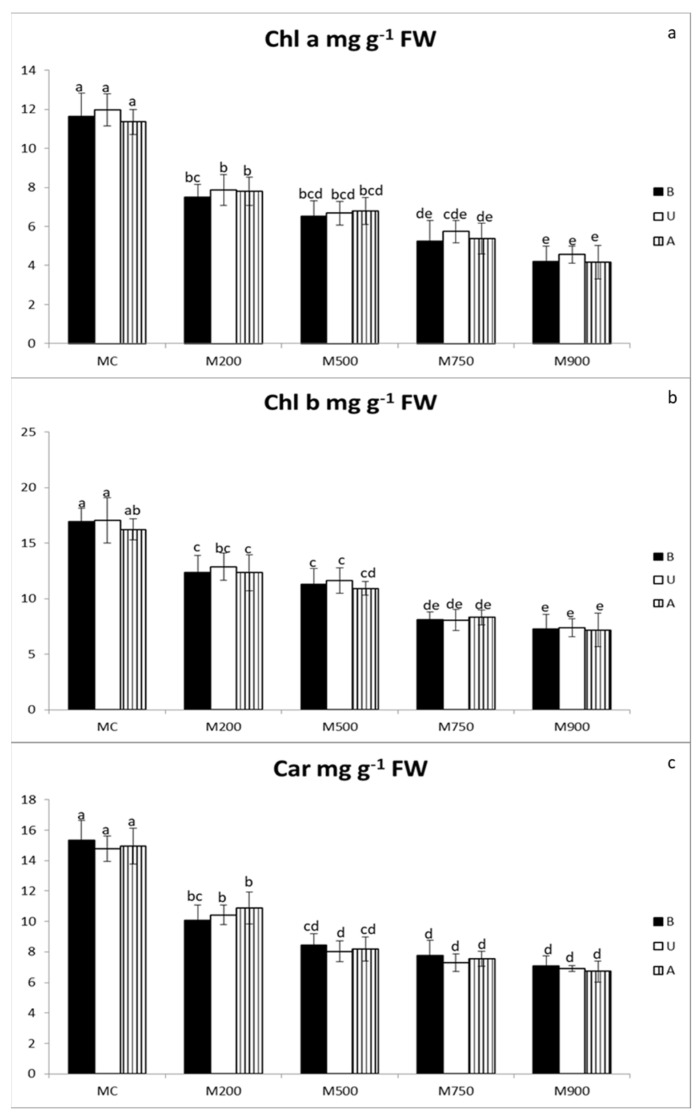
Chlorophyll a (**a**), chlorophyll b (**b**), and carotenoid (**c**) content in the leaves of three different miscanthus cultivars (B, U, and A) cultivated in soils with gradient TE concentrations (MC, M200, M500, M750, and M900). Values are presented as means ± SD. Different letters refer to significant differences between plants (Turkey HSD test, *n* = 5, *p* ≤ 0.05).

Photosynthetic pigments (Chl a, b, and car) (Figure 2a, b, and c, respectively) concentrations in miscanthus leaf tissues exhibited an inverse response pattern compared to the antioxidant enzymatic activities. Generally, their concentrations were negatively affected by TE contamination. However, the same two-level response was observed. Actually, the concentrations of the investigated photosynthetic pigments strongly decreased in plants grown in M200 soil compared to MC soil. Then, their contents in leaves slowly decayed in a concentration-dependent manner. No significant differences were observed among the miscanthus cultivars. 

Chl a concentration significantly declined as soil the contamination increased. The highest concentrations were observed in the leaves of the miscanthus cultivated in MC soil (11.63, 11.96, and 11.36 mg g^−1^ FW corresponding to miscanthus B, U, and A, respectively). The drop recorded 63.8, 61.9, and 63.2% in B, U, and A shoots cultivated in M900 soil. 

Chl b levels were 16.9, 17.1, and 16.2 mg g^−1^ FW in miscanthus B, U, and A, respectively cultivated in MC soil and decreased by 57.1, 56.7, and 55.7% in those cultivated in M900 soil. 

Maximal car concentrations were detected in the non-contaminated MC leaves (15.3, 14.8, and 14.9 mg g^−1^ FW corresponding to miscanthus B, U, and A, respectively). The reduction in M900 soil was by 54.0, 53.2, and 55.1% in B, U, and A cultivars, respectively.

### 3.5. Secondary Metabolites Quantification

**Figure 3:**
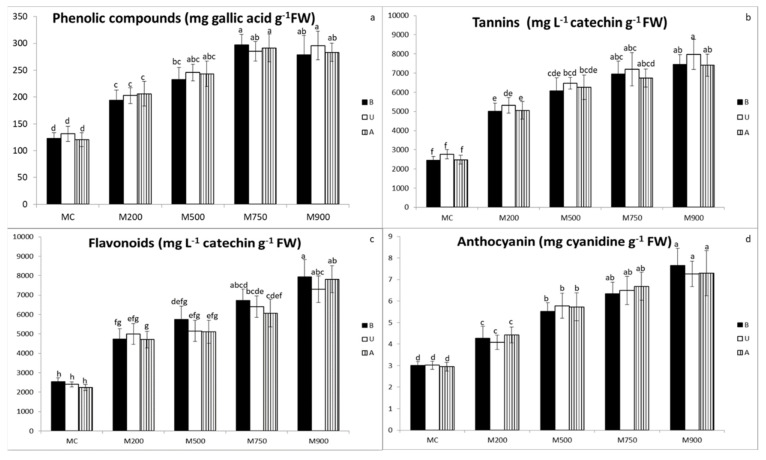
Phenolic compounds (**a**), tannin (**b**), flavonoid (**c**), and anthocyanin (**d**) concentrations in the leaves of three different miscanthus cultivars (B, U, and A) cultivated in soils with gradient TE concentrations (MC, M200, M500, M750, and M900). Values are presented as means ± SD. Different letters refer to significant differences between plants (Turkey HSD test, *n* = 5, *p* ≤ 0.05).

Secondary metabolite accumulations in miscanthus leaf tissues showed the same response patterns to soil TE concentration increases as the antioxidant enzymes. Globally, the concentrations of the investigated secondary metabolites (phenolic compounds, tannins, flavonoids, anthocyanins, Figure 3a, b, c, and d, respectively) strongly increased in plants grown in M200 soil compared to MC. Thereafter, the accumulation in leaves slowly continued in a concentration-dependent manner. In the four metabolites, no significant differences were observed among the three miscanthus cultivars.

Phenolic compounds levels gradually increased from 122.8, 131.3, and 120.8 mg gallic acid g^−1^ FW, corresponding in the leaves B, U, and A plants cultivated in the MC soil, to 297.0, 295.9, and 291.3 3 mg gallic acid g^−1^ FW, corresponding to B, U, and A cultivated in the M750. M900 and M750 soils respectively recorded 141.8, 125.4, and 141.3% accretion compared to background values. 

Tannin concentration increased as well with the increase in the contamination. Values recorded were 2444.6, 2765.3, and 2483.4 mg L^−1^ catechin g^−1^ FW in B, U, and A cultivars of MC soil, and increased by 204.3, 188.2, and 198.5% compared to background values in the plants of M900 soil.

Flavonoid quantity increased in the miscanthus leaves with soil contamination. Minimal inductions were detected in miscanthus leaves planted in MC soil (2541.1, 2400.1, and 2232.8 8 mg catechin L^−1^ g^−1^ FW in B, U, and A cultivars, respectively), and rose progressively, yielding 212.3, 204.3, and 249.9% augmentation compared to background values in those cultivated in M900 soil.

Anthocyanin results in the leaves of the three cultivars showed that the plants cultivated in MC soil recorded the lowest concentrations (3.0 mg cyanidin g^−1^ FW in the leaves of the three cultivars) and were boosted by 154.8, 140.7, and 147.3% compared to background values in B, U, and A plants, respectively, cultivated in M900 soil.

## 4. Discussion

The ability of *Miscanthus* × *giganteus* to thrive in contaminated areas was discussed in several papers, and it was nominated as a good candidate for phytostabilization and phytomanagement of severely contaminated areas [14,15]. Its positive impacts on decreasing metal bioaccessibility and restoring soil functionality via enhancing the soil biological parameters have been discussed in some previous works as well [6,20]. However, the experiments that evaluate TE impacts on miscanthus health are scarce and have never been undertaken for more than three months. In the previous work [24], after a full growing cycle on TE contaminated soils (T1), we demonstrated that miscanthus plants exhibited TE-induced stress (an increase in antioxidant enzymatic activities, a decrease in photosynthetic pigment content, and an enhancement of secondary metabolite accumulation). However, results also demonstrated that miscanthus plants were quite tolerant to TEs as no significant differences were observed between plants growing on different soils with an increasing gradient in TE concentrations. The aim of this discussion is not to examine the TE influence on *M.* × *giganteus* physiology which has been already discussed in the first part of this work [24], rather consider its TE tolerance between the two growing seasons, and to compare it with other miscanthus species. 

In the present study, at the end of the second growing cycle (T2), miscanthus exhibited a significant difference in response to the exposure to multiple TE concentrations. The decline in the tillers number in the contaminated soils in comparison to the non-contaminated ones expressed well the stressful cultivation environment (Table 2). Our data showed a clear decline in tillers numbers, up to 60% in the most contaminated in comparison to MC soil. On the other, in the first growing season, a decrease was observed only in the extremely contaminated soil (M900) and only with U cultivar [24]. Fernando and Oliveira [32] also demonstrated the negative impacts of soil contamination on miscanthus growth. However, on two soils multi-contaminated with TE, this impact was less intense, with a 33.3% decrease in the number of tillers per plant compared to control plants. On the other hand, in the present work, no significant differences were recorded concerning the plants’ height, unlike Fernando and Olivera [32], who showed a 21.6% decrease in the plants cultivated in contaminated soil. Guo et al. [22], as well, showed that the height of three miscanthus species (*M. sinensis*, *M. floridulus,* and *M. sacchariflorus)* decreased with an increase in Cd concentration (respectively 18.3, 17.1, and 12% shorter than their corresponding controls). Zhang et al. [23] observed an up to 47.4% decrease in the height of the *M. sacchariflorus* cultivated in Cd-spiked soil (100 mg kg^−1^). 

The observed negative impact of TE contamination on plant growth can be explained by several deleterious effects induced by the TEs at physiological and molecular levels [33,34]. Our results on antioxidant enzymatic activities clearly suggest that miscanthus is facing intense oxidative stress (Figure 1a–c). For each antioxidant enzyme (SOD, APX, GR), a strong activity increase was observed in M200 soil compared to the control soil (MC), followed by a dose-dependent incrementation. These results opposed the first growing season (T1), where no significant difference was observed between plants grown in contaminated soils [24]. In addition, the measured activities at T2 were on average 27.7% higher than at T1. However, our T2 results are in accordance with those of Guo et al. [22]. These authors showed a dose-dependent increase in the SOD, APX, and GR activities, as well as catalase and peroxidases activities. Nevertheless, the TE-induced increases were two to three times higher in hydroponic conditions. Conversely, a dose-dependent increase in SOD, APX, and peroxidase activities, followed by a significant decrease at the higher concentrations, was observed in *M.* × *giganteus* plants grown in hydroponic conditions [35] and in *M. sacchariflorus* grown on spiked soils with Cd [23]. 

In our experiment, this TE-induced oxidative stress coincided with a significant decrease in the photosynthetic pigments (Chl a, Chl b, and Car) in the three miscanthus cultivars. The response showed the same two-level pattern (Figure 2a–c). A strong decrease in pigment concentrations was observed in the M200 soil compared to the MC soil, followed by a slower but concentration-dependent decline. This concentration-dependent pigment concentration reduction was also demonstrated in *M. sinensis*, *M. floridulus,* and *M. sacchariflorus* exposed to Cd in hydroponic conditions [22] and *M. sacchariflorus* grown on Cd-spiked soils [23] but are in contradiction with our T1 results [24]. Moreover, in comparison with the first growing season results [24], the pigment concentration decays were higher at the end of the second growing season (on average, on the M900 soil, up to 62.9% in T2 vs. 37.2% in T1 for Chl a; 56.5% vs. 26.1% for Chl b; 54.1% vs. 32.4% for Car). These results are in accordance with the results of Guo et al. [22] who showed a total chlorophyll loss up to 57.7, 56.8, and 36.6% in *M. sinensis*, *M. floridulus,* and *M. sacchariflorus* plants cultivated in the most Cd-contaminated solution (200 µM), concomitant with, respectively, a 48.6, 44.8, and 20.8% decrease in carotenoids content. In the TE tolerant *M. sacchariflorus* grown on Cd-spiked soils, the decrease in Chl a, Chl b, and Car contents was up to 34.9, 26.9, and 18.4%, respectively [23]. 

Secondary metabolism plays an important role in plant TE tolerance and defense against oxidative stress [36]. However, no study has investigated the effect of TE on miscanthus secondary metabolism yet. In our experimental conditions, after one growing season, results showed that all the investigated secondary metabolite (phenolic compounds, tannins, flavonoids, anthocyanins) concentrations increased in response to TE stress [24]. However, no significant differences were observed between plants growing in the different contaminated soils. Conversely, at the end of the second growing season, our data demonstrated a clear concentration-dependent response to soil TE contamination (Figure 3a–d). The same two-level patterns, as already observed for antioxidant enzymes and photosynthetic pigments responses, were noticed for phenolic compounds, tannins, and flavonoids. Their concentrations strongly increased in plants grown in M200 soil compared to MC soil, and then slightly boosted in a concentration-dependent manner. Anthocyanin accumulation, however, in response to TE contamination did not exhibit this two-level pattern and was totally concentration-dependent (Figure 3d). Moreover, as already observed for the other biomarkers, the response intensity was significantly higher but for anthocyanin (in the M900 soil up to 128.8% at T2 vs. 39.7% at T1, for phenolic compounds; 197.0% vs. 40.2% for tannins; 222.1% vs. 59.1% for flavonoids: 147.6% vs. 204.6% for anthocyanins).

Altogether, our data clearly demonstrated that our miscanthus plants were subjected to important TE-induced stress. During the first growing season, the plants were able to tolerate the increasing concentrations of TEs. However, at the end of the second growing season (T2), the evaluated stress was concentration-dependent, and on average, two times higher compared to T1. Moreover, this stress was comparable with the level of stress observed in experiments growing several miscanthus species in hydroponic solutions or in spiked soils, which usually poorly reflect the field reality. These significant differences between T1, T2, and other experiments could be due to the differences in TE uptake. Indeed, at T2, Cd, Pb, and Zn concentrations in the plants cultivated in contaminated soils were up to, respectively, 2.2, 7.2, and 1.9 times higher than at T1. The T2 Cd concentrations were also comparable to Cd concentrations found in miscanthus grown in Cd-spiked soils [16] or in hydroponic solution enriched with Cd [35,37]. Despite the fact that extremely high concentrations of Cd in hydroponic solutions were used, and very important stress in *M. sinensis, M. floridulus,* and *M. sacchariflorus* leaves was observed, Guo et al. [22] measured lower Cd concentrations in their leaves. This could be explained by the very short experiment duration (16 days). 

However, these observed TE-induced stresses and impacts on miscanthus biomass are quite surprising in respect to the fact that miscanthus is supposed to be a stress-tolerant plant [14]. Moreover, these results are not in agreement with field experiments. Actually, no significant effects of TE contamination on *M.* × *giganteus* biomass have been demonstrated in in-situ experimental plots in the Metaleurop area for almost ten years (unpublished data). Despite the fact that 20 kg of soil per pot was used (which is a large quantity of soil compared to other experiments which do not use more than 12 kg of soil), these results suggested that we over-estimated the TE-induced stress and impacts after two growing seasons. Although *Miscanthus* × *giganteus* plants do not intensively transfer metals to their aerial biomass, the prolonged time of TE exposure (18 months) in a precise soil volume and quantity might suggest stronger TE absorption and transport from the roots to the aboveground parts and hence result in increased leaf TE concentrations [22,23,38]. The outcome obtained is in conformity with Pourrut et al. [33], who stated that the effects of contamination intensity on plants depended on the TE concentrations as well as their exposure duration.

The same conclusion can be drawn for hydroponic and spiked soil experiments. Indeed, several works have criticized these types of experiments and shed light precisely on TE mobility, availability, translocation, and accumulation in the plants in culture [39,40]. For example, Hamels et al. [41] showed that TE availability and toxicity to barley seedlings grown on field-contaminated soils were up to 30 times lower than that on corresponding spiked soils. Moreover, Kumar et al. [40] demonstrated that the Pb shoot and root concentrations in a *Talinum* variety grown in hydroponic solution gradually increased as a function of increased Pb exposure. The chlorophylls, carotenoids, and lipid peroxidation as well were negatively impacted on the Pb treatments compared to the control ones.

## 5. Conclusions and Perspectives

The current work is a part of a series of studies that aim to evaluate the efficiency of *Miscanthus* × *giganteus* plants in remediating highly TE contaminated soils. After proving the positive impacts of cultivating *M. giganteus* in highly contaminated soils by decreasing metal bioaccessibility and thus alleviating the risks to humans, restoring soil functionality, and improving its physicochemical and biological parameters, the results obtained in the present study showed that the miscanthus tillers number was negatively affected by the soil TE contamination. Moreover, the plant’s response to increased soil TE concentrations was expressed by a decline in the photosynthetic pigment (Chl a, Chl b, and car) levels on the one hand, and the progressive induction of the phenolic compounds, tannins, flavonoids, and anthocyanin on the other hand. In addition, the antioxidant enzymatic activities of SOD, APX, and GR were also elevated with the TE concentrations in the soil. It was also recognized that in comparison to the results of the first growing season, the plant response was intensified as a result of the longer TE exposure and the limited volume and quantity of soils presented in the pots. This outcome might pose an important question about the relevance of long-term pot experiments in simulating actual field conditions. Therefore, further in-situ investigations are recommended to legitimize the obtained results concerning the TEs impacts on the miscanthus plants.

## Figures and Tables

**Table 1:** Cd, Pb, and Zn pseudo total concentrations in the studied soils (mean ± standard deviation).

	MC	M200	M500	M750	M900
Cd (mg kg^−1^)	0.3 ± 0.0	3.8 ± 0.2	9.0 ± 0.2	13.5 ± 0.3	16.0 ± 0.3
Pb (mg kg^−1^)	37.3 ± 1.3	260.3 ± 2.0	528.6 ± 5.3	747.1 ± 16.9	898.6 ± 16.3
Zn (mg kg^−1^)	54.6 ± 3.1	388.0 ± 14.8	537.0 ± 10.9	906.0 ± 16.8	1116.0 ± 1.7

**Table 2:** Leaf TE concentrations of the cultivars (B, U, and A) in soils with a gradient TE concentration (MC, M200, M500, M750, and M900) at the end of the second growing period. Values are presented as means ± SD. Different letters refer to significant differences between plants (Turkey HSD test, *n* = 5, *p* ≤ 0.05).

		Cd (mg kg^−1^)	Pb (mg kg^−1^)	Zn (mg kg^−1^)
B	MC	0.4 ± 0.0 h	6.9 ± 0.8 f	37.2 ± 3.1 h
	M200	0.7 ± 0.1 g	12.6 ± 1.4 e	76.1 ± 5.3 f
	M500	1.4 ± 0.1 e	19.8 ± 1.7 cd	90.9 ± 4.8 de
	M750	2.4 ± 0.1 c	25.9 ± 3.8 b	114.7 ± 4.5 b
	M900	3.1 ± 0.1 a	31.0 ± 2.1 a	138.2 ± 4.1 a
U	MC	0.4 ± 0.0 h	5.9 ± 0.4 f	29.0 ± 3.7 h
	M200	0.7 ± 0.1 g	11.2 ± 2.0 ef	66.2 ± 4.4 g
	M500	1.1 ± 0.1 f	18.0 ± 1.7 d	82.2 ± 4.6 ef
	M750	2.1 ± 0.1 d	23.1 ± 1.9 bc	104.7 ± 4.0 c
	M900	2.7 ± 0.1 b	27.4 ± 3.0 ab	116.3 ± 3.3 b
A	MC	0.4 ± 0.0 h	7.0 ± 0.9 f	41.5 ± 6.0 h
	M200	0.7 ± 0.0 g	12.8 ± 1.1 e	77.1 ± 5.2 f
	M500	1.2 ± 0.1 e	19.5 ± 1.4 cd	93.5 ± 2.1 d
	M750	2.3 ± 0.2 c	25.6 ± 2.6 b	120.4 ± 4.3 b
	M900	3.1 ± 0.1 a	31.7 ± 3.9 a	140.7 ± 4.9 a

**Table 3:** Tiller numbers per pot, height, and stem diameter of the cultivars (B, U, and A) in soils with a gradient TE concentration (MC, M200, M500, M750, and M900) at the end of the second growing period. Values are presented as means ± SD. Different letters refer to significant differences between plants (Turkey HSD test, *n* = 5, *p* ≤ 0.05).

		Tillers Number	Height (cm)	Diameter (mm)
B	MC	16.3 + 1.5 bc	109.3 ± 9.1 a	9.3 ± 1.8 a
	M200	10.0 + 2.9 def	99.2 ± 4.3 ab	8.7 ± 1.2 a
	M500	9.4 + 2.3 def	97.6 ± 7.1 abc	9.0 ± 1.4 a
	M750	6.0 + 1.7 f	96.0 ± 5.9 abc	8.4 ±1.6 a
	M900	6.0 + 1.9 f	94.8 ± 4.1 abc	8.7 ± 1. a
U	MC	27.3 + 4.2 a	92.7 ± 5.9 abc	7.3 ± 0.7 a
	M200	12.0 + 1.2 bcd	87.6 ± 7.2 bc	8.2 ± 0.7 a
	M500	11.2 + 2.2 bcde	86.2 ± 4.1 bc	8.1 ± 0.6 a
	M750	9.0 + 2.8 def	86.0 ± 7.9 bc	7.8 ± 1.0 a
	M900	7.4 + 2.7 def	84.4 ± 5.4 c	8.3 ± 0.4 a
A	MC	17.0 + 2.0 b	106.3 ± 6.8 a	9.2 ± 0.3 a
	M200	10.8 + 2.6 cdef	100.2 ± 5.9 ab	8.7 ±0.8 a
	M500	10.0 + 2.3 def	98.8 ± 6.9 ab	8.7 ± 0.7 a
	M750	6.4 + 1.6 ef	97.2 ± 9.3 abc	8.4 ± 1.4 a
	M900	6.0 + 0.9 f	95.2 ± 9.2 abc	8.8 ± 1.1 a

## Data Availability

Data sharing is not applicable.
